# Effective Detection of Porcine Cytomegalovirus Using Non-Invasively Taken Samples from Piglets

**DOI:** 10.3390/v9010009

**Published:** 2017-01-12

**Authors:** Vladimir A. Morozov, Gerd Heinrichs, Joachim Denner

**Affiliations:** 1 Robert Koch Institute, 13353 Berlin, Germany; 2 Aachen Minipigs, 52525 Heinsberg, Germany; heinrichs.karken@web.de

**Keywords:** Aachen minipigs (AaMP), non-invasive sampling, porcine cytomegalovirus (PCMV), infection, detection, sensitivity, nested PCR, real-time PCR

## Abstract

Shortage of human organs forced the development of xenotransplantation using cells, tissues, and organs from pigs. Xenotransplantation may be associated with the transmission of porcine zoonotic microorganisms, among them the porcine cytomegalovirus (PCMV). To prevent virus transmission, pigs have to be screened using sensitive methods. In order to perform regular follow-ups and further breeding of the animals, samples for testing should be collected by low-invasive or non-invasive methods. Sera, ear biopsies, as well as oral and anal swabs were collected from ten 10-day-old Aachen minipigs (AaMP) and tested for PCMV using sensitive nested polymerase chain reaction (PCR) as well as uniplex and duplex real-time PCR. Porcine cytomegalovirus DNA was detected most frequently in oral and anal swabs. Comparison of duplex and uniplex real-time PCR systems for PCMV detection demonstrated a lower sensitivity of duplex real-time PCR when the copy numbers of the target genes were low (less 200). Therefore, to increase the efficacy of PCMV detection in piglets, early testing of oral and anal swabs by uniplex real-time PCR is recommended.

## 1. Introduction

Xenotransplantation using pig cells, tissues, or organs made enormous progress in recent years as an alternative for allotransplantation [1]. However, xenotransplantation may be associated with the transmission of porcine zoonotic microorganisms, including the porcine cytomegalovirus (PCMV).

Cells, organs, and tissues of pigs have been used in first preclinical and clinical xenotransplantation trials (for review see [2]) and it was clearly shown that in pig to non-human primate transplantation trials using transplants from PCMV-infected animals, replication of PCMV took place in the recipient [3,4]. In another case, despite the fact that PCMV was undetectable in a donor animal, the virus was transmitted with the heart transplant and actively replicated later in the non-human primate recipient [5]. In all cases, it remained unclear whether PCMV infected cells of the recipient. However, PCMV transmission reduced nearly three times the survival time of the transplant as demonstrated in two kidney xenotransplantation trials using organs from α-1,3-galactosytransferase knockout (GalT-KO) pigs into baboons [6] or cynomolgus monkeys [7]. Based on these and some earlier data, PCMV is considered a potential zoonotic pathogen [8,9,10,11].

PCMV is an enveloped virus with a double stranded DNA genome. It belongs to the *Herpesviridae* family, subfamily *Betaherpesvirinae*, genus *Roseolavirus*. The PCMV genome contains 128,367 nucleotide pairs, 79 open reading frames (ORFs), and 73 of those have promotors and eight genes coding for putative proteins with unique sequences and yet unknown functions [12]. The diversity of PCMV on a full genome level was not well investigated, since only one virus isolate from pulmonary alveolar macrophages was completely sequenced [12]. Porcine cytomegalovirus is genetically more closely related to human herpesviruses 6A and 6B (HHV-6A, B) and 7 (HHV-7), than to human cytomegalovirus (HCMV, HHV-5).

Porcine cytomegalovirus infection of pigs is endemic [13,14,15,16]. Virus transmission occurs horizontally through nasal and ocular secretions, milk, and urine. Data on transplacental infections are controversial [15,17,18,19]. In newborn and young piglets, the virus may cause inclusion body rhinitis and generalized infection. However, in adult animals PCMV is latent and the virus titer in body fluids and organs is very low or undetectable [15]. The low virus titer in the blood is a principal obstacle in PCMV diagnostic by PCR. Furthermore, at present it is still unknown whether PCMV infects human cells. Results of in vitro infection studies using co-cultivation of infected porcine cells with two established human cell lines [8] and primary fibroblasts [20] were controversial.

Pig blood and sera were most frequently used for routine PCMV testing. However, it was shown that in terms of PCMV, blood is not the most representative biological material [15]. Testing of organs for PCMV could be more informative, but the results are limited to the tested animals and do not provide a full-scale picture of virus spread in the herd. To improve systematic testing of pigs, there are several possibilities: early testing using non-invasive sampling methods, optimized DNA extraction for selected material, and highly sensitive methods of virus diagnostic. However, it was unknown what might be the best diagnostic sample material to be collected by non-invasive methods.

Previously, extensive screenings for the PCMV genome were performed in a variety of tissues and body fluids from farm animals of different ages using real-time polymerase chain reaction (PCR) [15,21]. These studies demonstrated that, in adult animals, the virus was not detected in blood, urine, feces, nasal, and oral swabs, but it was present in low copy number in the spleen and in peripheral blood mononuclear cells (PMBC). In contrast, PCMV was found in all examined organs in three out of four, three to five-week-old piglets, and in nasal and saliva swabs of one piglet, however the feces of all piglets were PCMV DNA negative [15]. When three week-old piglets were tested, a high virus load (>4 × 10^7^ copies/mg) was detected in the thymus [22]. However, other organs were not analyzed.

Here a comparative study of PCMV detection in selected biological sample material collected by low-invasive and non-invasive methods was performed with the goal to select negative animals for further breeding. A cohort of 10-day-old Aachen minipigs (AaMP) was analyzed by different PCR approaches using sera, ear biopsies and oral and anal swabs. Sensitive nested PCR and real-time PCR systems in uniplex and duplex versions were used for PCMV DNA detection and the sensitivity of the methods was compared. In addition, PCMV amplicones were sequenced in order to confirm that it was PCMV, to investigate putative viral variability and to exclude that there was contamination during PCR handling.

## 2. Materials and Methods

### 2.1. Animals and Examined Samples

Aachen minipigs are new minipig breeds produced in Aachen (Germany) for medical and pharmaceutical research. Production of animals is registered under protocol no. 27605370/0120349 (Veterinär- und Lebensmittelüberwachungsamt, Kreis Heinsberg, Germany).

Pregnant sows were kept separately in special bays. After birth, the piglets stayed with the mother for six weeks for breast feeding. Then, the animals were weaned and different litters were mixed to form groups of 20 animals. Testing at this time point is relevant to segregation strategies for animals destined for research purposes. The first microbiological characterization of randomly selected AaMP was performed recently [23].

In this study, a cohort of 10-day-old AaMP from five litters born from five parent pairs (five sows and four boars) was examined. Oral and anal swabs were collected using sterile cotton buds and shipped cooled together with blood and ear biopsies (about 3 mm^2^), and processed immediately after delivery.

### 2.2. DNA Extraction

To obtain comparable results, all DNA extraction procedures were performed using the Quick-DNA Universal kit (Zymo Research, The Epigenetics Company, Irvine, CA, USA). Material from cotton swabs were eluted in 200 μL of phosphate buffered saline (PBS) and 100 μL were used for DNA extraction that was repeated twice. To reduce the amount of PCR inhibitors, DNA samples from anal swabs were additionally purified using One Step PCR Inhibitor Removing kit (Zymo Research). DNA from ear biopsies was extracted from 10 to 15 mg of the tissue. For complete sample digestion, proteinase K treatment was extended to 18 h. DNA extraction from sera was performed two times, using each time 100 μL and the DNA was eluted in a final volume of 35 μL. The DNA specimens were quantified on NanoDrop spectrophotometer ND-1000 (Thermo Fisher Scientific, Inc., Worcester, MA, USA).

### 2.3. Conventional Polymerase Chain Reaction and Nested Polymerase Chain Reaction

Primers and probes for PCR systems (Table 1) were synthesized by Sigma-Aldrich (Munich, Germany). The Gibbs free energy change (ΔG) and melting temperature (T_m_) for oligonucleotides hair pins were estimated using Oligo Analyzer 3.1 (Integrated DNA technologies, Coralville, IA, USA; Table 1). Estimation of the PCR sensitivity was reported earlier [24].

The nested PCR was performed with the GoTaq Green master mix according to the protocol of the supplier (Promega, Madison, WI, USA) using primers F1 and R1. After initial denaturation at 95 °C for 2 min 40 cycles of denaturation at 94 °C for 30 s, annealing at 58 °C for 30 s and extension at 70 °C for 40 s were performed. The length of the amplicon after the first-round PCR was 350 bp. Using one microliter from the first reaction the second PCR round of 35 cycles was performed with primers F2 and R2 (annealing temperature 54 °C) and extension was reduced to 30 s. The length of the amplicon after the second-round PCR was 206 bp.

### 2.4. Cloning and Sequence Analysis of the Amplicons

Polymerase chain reaction amplicons were ligated into the pCR2.1-TOPO vector according to the protocol of the supplier (Invitrogen Life Technologies, Carlsbad CA, USA). Mix & Go competent cells (Zymo Research, The Epigenetics Company, Irvine, CA, USA) were transformed with the constructs and plated on lysogeny broth (LB) agar/ampicillin dishes for 18 h at 37 °C. Colonies were collected and amplified in LB/ampicillin medium overnight at 37 °C. Plasmids were isolated using PureYield plasmid miniprep system (Promega, Madison, WI, USA) and sequenced in both directions using primers from the cloning kit and BigDye terminator v.3.1 cycle sequencing kit (Applied Biosystems, Darmstadt, Germany).

### 2.5. Real-Time Polymerase Chain Reaction

**Figure 1:**
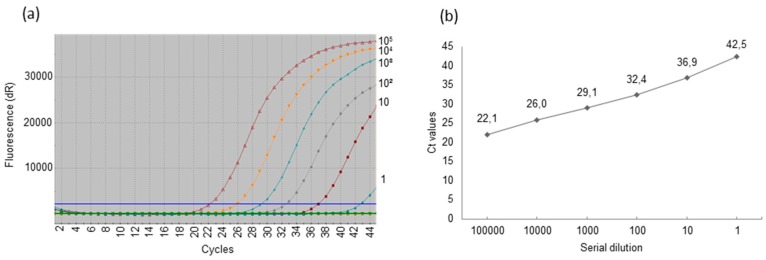
Estimation of the sensitivity of the real-time polymerase chain reaction (PCR)—a representative result. Serial 10-fold dilutions of the reference plasmid containing the PCMV *DNA polymerase* (*DNApol*) gene were used for quantification. (**a**) Amplification plots. dR: reporter signal normalized to the fluorescent signal of the fluorophore ROX (Rn) minus the base line; (**b**) Standard curve. The reference plasmid was diluted in nuclease-free H_2_O containing 100 ng/μL of salmon sperm DNA. Cycle threshold (Ct) is inversely proportional to the amounts of target in the reaction.

DNA (200 ng) was extracted from ears, oral and anal swabs, and 50 ng of DNA was extracted from sera and used for testing. The real-time PCR was performed using SensiFast probe no ROX kit according to supplier recommendations (Bioline GmbH, Luckenwalde, Germany). Primers and probes are listed in Table 1. The reaction was performed in 20 μL. The PCR conditions were as follows: enzyme activation for 5 min at 95 °C was followed by 45 cycles of denaturation at 95 °C for 10 s, annealing at 59 °C for 20 s, and extension at 62 °C for 30 s. Design of the reference plasmid was described previously [24]. For the target quantification experiments were accompanied by a serial 10-fold dilution (1x10^5^, 1x10^4^, 1x10^3^, 1x10^2^, 1x10^1^ and two or one copy) of the reference plasmid, example is shown (Figure 1). The sensitivity of the uniplex real-time PCR system was 1–2 copies per reaction and the estimated efficiency of the real-time PCR was between 0.98 and 1.02. The real-time PCR was performed as uniplex or as duplex PCR, using the porcine *cyclophilin A* gene as a housekeeping control with primers and probe described previously [25]. Reaction was performed in a Stratagene MX3005P system (Agilent Technologies, Santa Clara, CA, USA). Each sample was tested in duplicates, once using uniplex real-time PCR and once using duplex real-time PCR. In addition for confirmation, oral samples were tested using two uniplex real-time PCRs and one duplex real-time PCR. Serum samples were tested using two uniplex real-time PCRs and one duplex real-time PCR. Anal swabs were tested using three uniplex real-time PCRs. Ear biopsies were tested using two uniplex real-time PCRs and one duplex real-time PCR.

### 2.6. Software

The Basic Local Alignment Search Tool (BLAST) program from the National Center for Biotechnology Information (NCBI) [26], was used for search. Oligo analyzer 3.1 (Integrated DNA technologies, Coralville, IA, USA) was used to estimate parameters of the oligonucleotides. The sequence alignments were performed using software package Lasergene Version 10 (DNASTAR, Inc., Madison, WI, USA).

## 3. Results

### 3.1. Characterization of DNA from Samples Collected by Non-Invasive and Low-Invasive Methods

To analyze the PCMV virus load, samples collected by non-invasive or low-invasive methods from 10-day-old AaMP were analyzed. DNA was extracted from all samples and tested by PCR specific for the PCMV *DNA polymerase* (*DNApol*) gene. Two forms of viral DNA might be expected in these samples, DNA-associated with virus particles, and viral DNA from the nucleus and cytoplasm of the infected cells. Since swabs contain both viral DNA from infected cells and cell-free viral DNA from saliva or anal mucosal fluid, a comparison of delta cycle threshold-ΔCt (Ct of the detected target minus Ct of the housekeeping gene), that are frequently used in real-time PCR for quantification, is not applicable. It should be emphasized, that the anal swabs contained only traces (if any) of stool. To eliminate possible PCR inhibitors, the DNA underwent additional purification steps as described in Material and Methods section.

An equal amount (200 ng) of DNA from ear biopsies and oral and anal swabs was tested in each reaction. Since the amount of cellular DNA in sera was low, only 50 ng of DNA were tested in every experiment. The presence and integrity of cellular DNA was estimated by uniplex real-time PCR using the porcine *cyclophilin A* gene (Table 2). Tests were performed in duplicates. Since the external ear contains predominantly cartilaginous tissues (ear chondrocytes were the most abundant cells, representing 10% of the total ear mass), the amount of extracted DNA was relatively low compared to the amount of DNA that might be extracted using the same amount of material from other organs. However, among all samples examined, the highest amount of cellular DNA was extracted from the ear biopsies and the lowest, as expected, from sera. Mean values for the extracted DNA were the following: ear biopsies - 95 ng/µL; oral swabs - 57 ng/µL, anal swabs - 66 ng/µL and sera - 12 ng/µL.

The amount of saliva in oral swabs was not possible to estimate, explaining the high Ct values (Table 2) of a housekeeping gene despite relatively high amounts of DNA. It is known, that cellular DNA undergoes fragmentation in saliva because of nuclease activity [27]. As a result, a significant amount of short oligonucleotides are present in oral swab samples. These oligonucleotides can increase the overall optical density reading, but because of their size they cannot be detected by real-time PCR targeting a house-keeping gene. Most likely that was the reason why the Ct values of a house-keeping gene, when testing the DNA from oral swabs, were relatively high. The mean Ct values of *cyclophilin A* in sera and oral swabs were close, and those of the anal swabs and ear biopsies were nearly identical (Table 2), therefore they were adequate to make a judgement.

### 3.2. Comparative Analysis of DNA Samples from Aachen Minipigs by Nested Polymerase Chain Reaction

**Figure 2:**
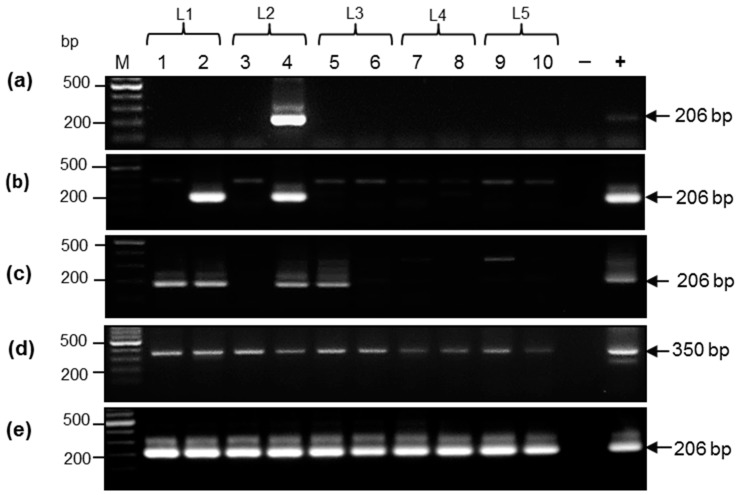
Detection of PCMV *DNApol* gene by nested PCR in different biological samples from Aachen minipig (AaMP) piglets. Animals from five litters (L) were examined. Brackets mark animals from the same litter. (**a**) DNA from sera; (**b**) DNA from anal swabs; (+) positive control 50 copies of a plasmid [23]; (**c**) DNA from ears; (**d**) DNA from oral swabs (first round PCR); (**e**) DNA from oral swabs (second round PCR). (+), positive control in (**b**–**e**) is DNA from a PCMV infected pig. (−), master mix without DNA. Positions of the amplicons are marked with arrow heads. Position of size markers (500 and 200 bp) are given on the left.

DNA extracted from all biological samples was initially tested using conventional nested PCR using primers targeting the PCMV *DNApol* gene. Positive samples were revealed in all types of specimens, but the number of positive samples differed significantly (Figure 2). Samples from piglet #4 were all positive and three out of four samples from piglet #3 were positive too. Most frequently, PCMV DNA was detected in oral swabs. All 10 DNA samples from oral swabs were positive in the first PCR round, indicating that the initially present target load was above 100 gene equivalent (g.e.)/reaction [24]. The high PCMV load in oral swabs was further confirmed by real-time PCR (see Section 3.4). Among DNA samples, 4 out of 10 ear biopsies were also tested positive. Finally, when sera was tested, only 1 out of 10 samples was found positive, indicating that the PCMV DNA load in sera is lower compared to other specimens. Some extra bands were detected above the expected amplicon after nested PCR (Figure 2B,C). These amplicons were sequenced, but they were all not virus-specific.

### 3.3. Porcine Cytomegalovirus Sequences Detected in Piglets

**Figure 3:**
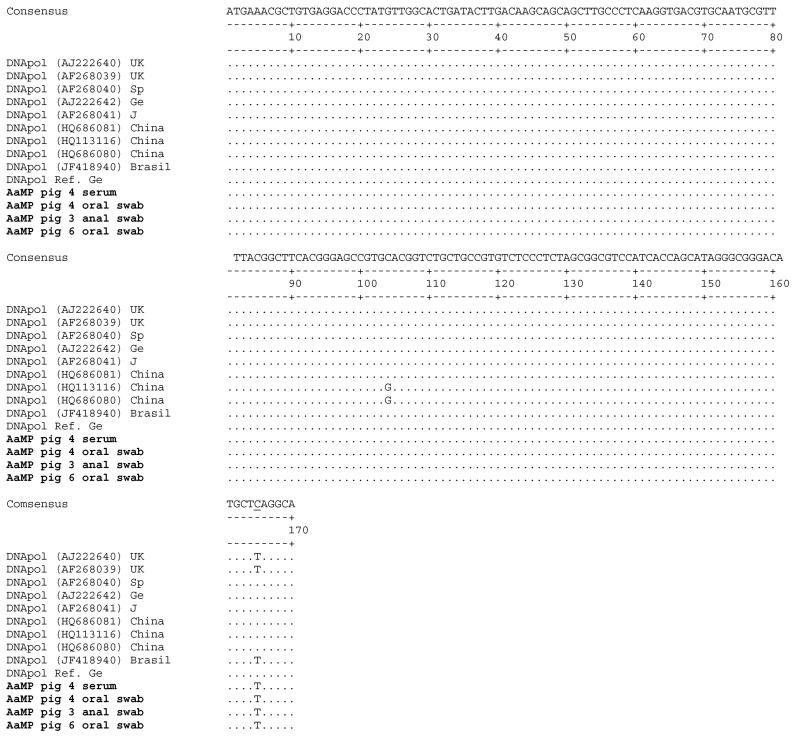
Alignment of the PCMV *DNApol* gene sequences amplified by nested PCR using DNA from serum and oral and anal swabs of three AaMP pigs. Each PCMV sequence detected in AaMP represented a consensus of five cloned sequences. “DNApol Ref. Ge” is a reference sequence amplified from a German landrace pig [23]. Genbank accession numbers are given in brackets. UK, United Kingdom; Sp, Spain; Ge, Germany; J, Japan. The substitution C165T is underlined.

The 206 bp PCR amplicons from piglets #3 (anal swabs), #4 (serum and oral swabs), and #6 (oral swabs) were cloned and sequenced and after sequence edition, a 170 bp nucleotide fragment was aligned with a set of PCMV *DNApol* gene sequences from pigs of different geographical origin (United Kingdom, Spain, Germany, Japan, China, and Brazil) (Figure 3). All amplicons from serum and oral and anal swabs showed full identity in-between. Thus, it is very likely that the same virus is circulating in all examined animals. Compared to the reference plasmid, a single nucleotide mismatch (substitution C165T) was found, but does not induce an amino acid change. However, this indicates that the sequenced viruses do not represent a contamination and that the primers are still adequate.

### 3.4. Detection of Porcine Cytomegalovirus by Real-Time Polymerase Chain Reaction and Comparison of the Sensitivity of Uniplex and Duplex Systems

The efficiency of a duplex PCR specific for the PCMV *DNApol* gene and porcine cyclophilin A gene was compared with that of a uniplex PCR specific for the PCMV *DNApol* gene. Duplex PCR was previously successfully used to investigate multiple virus infections [28]. Equal amounts of the same biological samples from AaMP were analyzed using both uniplex and duplex PCR systems (Table 3).

More PCMV-positive samples were detected by real-time PCR. To simplify the quantification based on Ct counts, the positive samples were divided into five groups: very strong (Ct 29 or lower; above 1 × 10^3^ g.e./reaction); strong (Ct 29–30; 1000–500 g.e./reaction); moderate (Ct 31–35; 250–50 g.e./reaction); weak (Ct 36–37; 25–10 g.e/reaction); and single copy positive (Ct 38–43 g.e./reaction; 5–1 g.e./reaction). In our hands, using SensiFast Probe No-Rox kit and a uniplex real-time PCR system, a single copy target was detectable in 3/5 tests at Ct 42–43. Note, that detection of single copies (<5) by real-time PCR is stochastic.

The positivity of all DNA samples from 10 oral swabs as detected by conventional PCR was further confirmed by uniplex real-time PCR (Table 3 and Table 4). All samples were rated as ‘very strong’ positive. Nine samples were also positive and comparably strong when tested in duplex PCR. Eight anal swabs samples were positive too, but all positive reactions were rated from ‘low’ to ‘single copy positive’. Of the sera samples, 6 of 10 were positive and rated as ‘low positive’ or ‘single copy positive’. It should be emphasized that none of the anal swabs and sera samples were positive when tested in duplex real-time PCR. Interestingly, six ear biopsies were positive in uniplex real-time PCR and three of them were positive in duplex PCR while the virus loads were very diverse, ranging from ‘strong’ positive to a ‘single-copy’ positive.

The comparison of two real-time PCR systems demonstrated a lower sensitivity of the duplex real-time PCR system (Table 3). However, when the amount of the target was above 200 g.e./reaction, both systems were equally sensitive. The uniplex PCR demonstrated a superior sensitivity when the amount of target was less than 100–200 g.e./reaction (>Ct 34). Further on, when using the duplex real-time PCR system, the targets could not be detected if the amount is below 15 copies/reaction (>Ct 35 or ‘low positive’ samples). Thus, the duplex real-time PCR used in this study was successful when hundreds or more copies of the main target per reaction were present.

## 4. Discussion

Despite substantial progress in xenotransplantation [1] the microbiological safety remains a still unresolved problem. Whereas acute viral infection could be detected in the donor pigs by clinical manifestations, some infections including PCMV are symptomless and may become chronic and therefore difficult to detect. Porcine cytomegalovirus is easily transmitted horizontally, but vertical transmission might not be excluded [15,17,18]. Porcine cytomegalovirus infection is ubiquitous and generally causes mild disease in pigs younger than six weeks [13,18]. In contrast, in adult pig PCMV is latent and if using only blood for analysis it is difficult to detect. The virus reservoir during latency is not clearly defined, but it likely includes immune cells. Transmission and replication of PCMV was observed in several preclinical xenotransplantations with a serious impact on the recipient [4,5,6,7].

Previously, an extended screening for PCMV in farm animals was performed using sensitive nested PCR, real-time PCR and serologic tests. Analyses of organs, blood, urine, feces, and oral and nasal swabs of adult pigs revealed PCMV predominantly in the spleen and lungs, while in body fluids, and nasal and oral swabs the virus was not detected [15]. In three out of four, three to five-week-old piglets, the virus was detected in blood and in the majority of the tested organs, but in only one animal was it detected in nasal and oral eluates [15]. However, it should be emphasized that DNA was not extracted from nasal and saliva eluates, but the aliquots were directly used in the PCR reaction after sample boiling. Though, it cannot be excluded that protein aggregates interfered with the Taq polymerase and significantly (if not completely) inhibit the amplification. As a consequence, false-negative results might be expected. No virus was detected in feces from piglets, but the authors did not indicate whether DNA was purified from PCR inhibitors that are associated with this type of samples. Interestingly, the same group reported that all DNA samples of blood from 1 to 14-day-old piglets were negative [15]. This result is close to what we observed analysing sera from 10-day-old AaMP. In the examined group, only 1 in 10 animals was found positive by nested PCR. In addition, evaluation of the prevalence of PCMV in farm animals older than six months in Brazil indicated that virus detection in the spleen was seven times more efficient than that in sera [29].

Sampling of blood and ear biopsies is a stressful procedure, especially for piglets. Nevertheless, blood and sera remained the most frequently used biological samples for PCMV testing in commercial farms and in facilities producing multitransgenic animals for xenotransplantation. Blood and ear biopsies were obtained by low-invasive methods, since the animals are not sacrificed. Here non-invasive samples such as oral and anal swabs have shown to be more favorable. Other samples such as urine, feces, and vaginal swabs have to be tested in future.

Early testing of piglets using oral swabs might represent the most efficient strategy to detect PCMV. Oral swabs may contain both DNA from virus particles and PCMV DNA from infected epithelial cells. However, low stability of free DNA in saliva means that mostly DNA from viral particles was detected in these samples. The viral DNA load in oral swabs was estimated as 5 × 10^3^–1 × 10^5^ g.e./µg and it was about two orders of magnitude higher than that detected in other samples (Table 5).

It should be emphasized that the oral swabs were obtained from suckling AaMP piglets and milk is considered to be a source of PCMV infection. Earlier, it has been shown that early weaning prevents virus transmission [4]. Thus, it cannot be excluded that cell-free PCMV or PCMV-infected epithelial cells from the milk contributed to the positive reaction observed with oral swabs. In this regard, it remains to be determined if PCMV from milk can infect and actively replicate in oral epithelial cells of piglets soon after birth. It is worth mentioning that analysis of porcine milk for PCMV infection has not been performed and, in this study, milk was not available for investigation. In this regard, it cannot be excluded that the efficacy of detecting PCMV positive oral swabs from fatteners or adult pigs might be lower.

Oral swabs from all piglets were PCMV DNA-positive; however, virus positivity in other samples varied. All samples from four animals (#4, #8, #9, and #19) were positive. Interestingly, the lowest frequency of PCMV detection was found for sera, a material that is frequently used for PCMV diagnostic. Analysis of ear biopsies showed a surprising result. A high number of positive animals was detected, including animals which serum DNA samples were PCMV DNA negative (#1, #2, and #5). Another unexpected result was a significant difference in PCMV DNA load (Table 4). For example, the highest PCMV DNA load was detected in piglet #2, it was 10–200 times above the level in the remaining six PCMV-positive animals. One explanation for the high virus load may be based on the aggressive behavior of the piglets within the first week of life, when a teat order and dominance hierarchy is established. Piglets may bite and chew ears of the others and contaminate the ears with saliva. In this regard, contaminated ear biopsies might demonstrate a significantly higher virus load in real-time PCR compared to the non-contaminated ones.

In this study, we compared duplex and uniplex real-time PCR as diagnostic tools for PCMV. This issue had been addressed earlier when analyzing conventional multiplex PCR systems for viral diagnostic [30]. In particular, problems with primer interference resulting in a reduction of the sensitivity in multiplex versus uniplex PCR were discussed. Limitations of duplex real-time PCR methods in detection of low copies of target genes five to seven orders of magnitude lower than the housekeeping gene were reported [25].

Here we demonstrated that compared to the sensitivity of the uniplex real-time PCR that of the duplex real-time PCR was lower. For example, samples that when analyzed by uniplex real-time PCR contained below 15 g.e./reaction were found negative when tested by duplex real-time PCR. Since the amount of the housekeeping gene compared with that of the target viral gene may differ by more than five to six orders of magnitude, proportional decreasing the concentration of the primers targeting the housekeeping gene would not solve the problem. Thus, to avoid false-negative results in diagnostic testing, it is useful to perform separate PCR reactions: one for the detection of the target gene, and another for the control housekeeping gene. However, it cannot be excluded that other duplex real-time PCR systems might perform better and be more sensitive in simultaneous detection of the housekeeping gene and the main target gene present at very low copy numbers.

## 5. Conclusions

Porcine cytomegalovirus DNA was detected in all ten 10-day-old piglets. Most frequently PCMV DNA was found by real-time PCR in oral (10 of 10 animals) and anal swabs (7 of 10 animals). In comparison, only 6 of 10 serum samples were positive. Based on the results described, several proposals on how to improve screening of piglets for PCMV infection can be made. First, piglets should be tested as soon as possible after birth, since the virus titer might be high and easy to detect. Second, to avoid stress and allowing further breeding, sampling should be performed by non-invasive means, for instance using oral and anal swabs. Simultaneous testing of both oral and anal samples may be a diagnostic advantage. Third, DNA extraction procedure should be optimized and only highly sensitive diagnostic method should be used for virus detection. Non-infected animals should be kept separately to prevent de novo infection. Finally, pigs used for xenotransplantation should be tested immediately before the organ transplantation.

## Figures and Tables

**Table 1:** Primers and probes.

Primers (nt)	Sequence (5′-3′)	T_m_ (°C)	Oligonucleotide Length	Homo-Dimers. ΔG below −7 kcal/mol	T_m_ Hairpin (°C)
**Conventional Polymerase Chain Reaction (PCR)**					
F1 (63–81) *	ACGGGGATCGACGAGAAAG	66.4	19	none	49
R1 (412–390) *	CTAGACGAGAGGACATTGTTGAT	61.1	23	none	29
F2 (182–201)*	GAAGAGAAAGGAAGTGAAGG	57.1	20	none	None
R2 (386–368) *	GTCACTCGTCTGCCTAAGC	60.2	19	none	30
**Real-time PCR**					
Fr-t (279–299) *	AATGCGTTTTACAACTTCACG	61.5	21	none	29
Rr-t (373–354) *	CTGAGCATGTCCCGCCCTAT	67.4	20	none	19
Probe (331–350) *	6FAM-CTCTAGCGGCGTCCATCACC-BHQ	69.2	20	none	17
F cyclophilin A (174–196) **	TGCTTTCACAGAATAATTCCAGGATTTA	59.1	28	none	23
R cyclophilin A (250–230) **	GACTTGCCACCAGTGCCATTA	61.7	21	none	22
Probe cyclophilin A (205–228) **	Cy5-TGCCAGGGTGGTGACTTCACACGCC-BHQ	67.1	25	none	44

* Nucleotide position of primers and probe for porcine cytomegalovirus (PCMV) amplification (GenBank accession no: AJ222640) [24]; ** Nucleotide position of primers and probe for cyclophilin A amplification (GenBank accession no: FN401368) [24]; T_m_: melting temperature; 6FAM: 6-carboxyfluorescein; BHQ: black hole quencher; Cy5: cyanine 5.

**Table 2:** Comparison of host DNA load in samples from piglets using real-time PCR using *cyclophilin A* as housekeeping gene. Samples were tested in duplicates. Mean Ct values are given.

Piglet #/Material	Sera	Anal Swabs	Ears Biopsies	Oral Swabs
1	29.7	22.2	22.3	26.1
2	29.6	23.4	24.6	24.3
3	27.8	21.5	23.0	25.5
4	26.8	21.8	22.0	25.5
5	27.7	21.6	20.5	28.5
6	27.8	21.7	20.7	26.4
7	28.3	21.9	21.3	28.5
8	28.3	22.3	23.5	28.0
9	28.2	25.6	21.6	26.7
10	27.1	23.6	21.0	25.9
Mean values:	28.13	22.56	22.05	26.54
Copies/reaction:	6 × 10^3^	5 × 10^6^	5 × 10^6^	2 × 10^4^
Copy/µg:	1.2 × 10^5^	2.5 × 10^7^	2.5 × 10^7^	1 × 10^5^

**Table 3:** Detection of PCMV in AaMP: comparative sensitivity of uniplex and duplex real-time PCR detection systems (mean Ct values are given, the samples were tested in duplicates).

Piglet #	Sera Uniplex Duplex		Oral Swabs Uniplex Duplex		Ears Biopsies Uniplex Duplex		Anal Swabs Uniplex Duplex	
1	Neg.	Neg.	30.0	30.0	35.8	Neg.	Neg.	Neg.
2	Neg.	Neg.	27.5	28.2	30.9	31.8	39.7	Neg.
3	38.6	Neg.	29.2	30.9	Neg.	Neg.	Neg.	Neg.
4	36.6	Neg.	28.4	30.8	39.4	Neg.	37.0	Neg.
5	Neg.	Neg.	27.3	29.1	34.0	41.3	36.2	Neg.
6	37.1	Neg.	28.1	28.6	Neg.	Neg.	41.3	Neg.
7	Neg.	Neg.	30.1	31.1	Neg.	Neg.	Neg.	Neg.
8	40.1	Neg.	29.4	31.4	32.3	39.7	39.5	Neg.
9	35.2	Neg.	26.1	26.9	35.2	Neg.	42.0	Neg.
10	37.2	Neg.	30.6	Neg.	43.0	Neg.	40.4	Neg.

Neg.: negative result.

**Table 4:** Virus load in examined samples and comparative sensitivity of nested and uniplex real-time PCR. Mean Ct values were given.

Piglet #	Sample	DNA Load (ng)	PCR	Nested PCR	Uniplex Real-Time PCR (Ct Values)	G.e./Reaction
1	Serum	50	Neg.	Neg.	Neg.	Neg.
	Anal swab	200	Neg.	Neg.	Neg.	Neg.
	Oral swab	200	+	+	30.0	~10^3^
	Ear biopsies	200	Neg.	+	35.8	~40
2	Serum	50	Neg.	Neg.	Neg.	Neg.
	Anal swab	200	Neg.	+	39.7	~2–5
	Oral swab	200	+	+	27.5	10^4^
	Ear biopsies	200	Neg.	+	30.9	~10^3^
3	Serum	50	Neg.	Neg.	38.6	~10
	Anal swab	200	Neg.	Neg.	Neg.	Neg.
	Oral swab	200	+	+	29.2	~2 × 10^3^
	Ear biopsies	200	Neg.	Neg.	Neg.	Neg.
4	Serum	50	+	+	36.6	~20
	Anal swab	200	Neg.	+	36.9	~20
	Oral swab	200	+	+	28.4	~7 × 10^3^
	Ear biopsies	200	Neg.	+	39.4	~2–5
5	Serum	50	Neg.	Neg.	Neg.	Neg.
	Anal swab	200	Neg.	Neg.	36.2	~20
	Oral swab	200	+	+	27.3	~10^4^
	Ear biopsies	200	Neg.	+	34.0	10e2
6	Serum	50	Neg.	Neg.	37.1	10
	Anal swab	200	Neg.	Neg.	41.3	~1–2
	Oral swab	200	+	+	28.1	~7 × 10^3^
	Ear biopsies	200	Neg.	Neg.	Neg.	Neg.
7	Serum	50	Neg.	Neg.	Neg.	Neg.
	Anal swab	200	Neg.	Neg.	Neg.	Neg.
	Oral swab	200	+	+	30.1	10^3^
	Ear biopsies	200	Neg.	Neg.	Neg.	Neg.
8	Serum	50	Neg.	Neg.	40.1	~2
	Anal swab	200	Neg.	+(weak)	39.5	~2
	Oral swab	200	+	+	30.4	10^3^
	Ear biopsies	200	Neg.	Neg.	40.1	~2
9	Serum	50	Neg.	Neg.	35.2	~30
	Anal swab	200	Neg.	Neg.	41.9	~1–2
	Oral swab	200	+	+	26.1	2 × 10^4^
	Ear biopsies	200	Neg.	Neg.	35.2	~40
10	Serum	50	Neg.	Neg.	37.2	~10
	Anal swab	200	Neg.	Neg.	40.4	~2
	Oral swab	200	+	+	30.6	10^3^
	Ear biopsies	200	Neg.	Neg.	38.1	~5

G.e.: genome equivalent.

**Table 5:** Summary. PCMV detection by different PCR methods and estimation of viral DNA load by uniplex and duplex real-time PCR.

Samples/Methods	PCR (Positive/Total)	Nested PCR (Positive/Total)	Uniplex Real-Time PCR (Positive/Total)	Duplex Real-Time PCR (Positive/Total)	G.e., Detected
Sera	1/10	1/10	6/10	0/10	10–150/mL
Anal swabs	0/10	3/10	7/10	0/10	5–100/μg
Oral swabs	10/10	10/10	10/10	9/10	5 × 10^3^–1 × 10^5^/μg
Ear biopsies	0/10	1/10	7/10	3/10	25–5 × 10^3^/μg
Total	11/40	15/40	30/40	12/40	
